# Early diagnosis and staging of paraquat-induced pulmonary fibrosis using [^18^F]F-FAPI-42 PET/CT imaging

**DOI:** 10.1186/s13550-024-01118-1

**Published:** 2024-06-18

**Authors:** Dimei Zhang, Yusheng Shi, Jiangwei Kong, Na Chen, Guiting Li, Mingfang Wang, Guoxia Zhang, Chuangyan Zhai

**Affiliations:** 1 Guangdong Provincial Key Laboratory of Tropical Disease Research, School of Public Health, Southern Medical University, Guangzhou, 510515 China; 2 Department of Nuclear Medicine, Nanfang Hospital, Southern Medical University, Guangzhou, 510515 China; 3 School of Forensic Medicine, Southern Medical University, Guangzhou, 510515 China; 4 Department of Radiation Oncology, Zhuhai People’s Hospital, Zhuhai Hospital Affiliated with Jinan University, Zhuhai, 519000 China; 5 Department of Pathology, Guangdong Women and Children Hospital, Guangzhou, 511400 China; 6 Research and Development Center, Guangdong Huixuan Pharmaceutical Technology Co., Ltd., Guangzhou, 510765 China

**Keywords:** [^18^F]F-FAPI-42, PET/CT, Pulmonary fibrosis, Early diagnosis, Disease staging

## Abstract

**Background:**

Paraquat (PQ) -induced pulmonary fibrosis poses a significant medical challenge due to limited treatment options and high mortality rates. Consequently, there is an urgent need for early diagnosis and accurate staging to facilitate appropriate treatment strategies. In this study, we assessed the diagnostic potential of [^18^F]F-FAPI-42 PET/CT imaging for early detection and disease staging in a rat model of PQ-induced lung fibrosis.

**Methods:**

After administering 80 mg/kg of PQ orally to Sprague-Dawley rats, we intravenously injected 3-3.5 MBq of [^18^F]F-FAPI-42 on day 7, 14, and 21 post-dosing. Dynamic PET/CT imaging was carried out for one hour immediately after the administration of [^18^F]F-FAPI-42. Subsequently, the lung tissues were collected for Hematoxylin and Eosin (HE) staining, Masson’s trichrome staining, and NOTA-FAPI-04-MB fluorescent probe staining. Data analysis was performed using the Imalytics preclinical software, and the mean standardized uptake value (SUV_mean_) was calculated.

**Results:**

PET signals revealed that in areas with evident lesions on CT, the SUV_mean_ on day 14 was significantly higher than on day 7 and 21, indicating that changes in fibrosis activity levels contribute to the staging of pulmonary fibrosis. Additionally, the NOTA-FAPI-04-MB fluorescent probe staining also demonstrated the most pronounced probe uptake on day 14. In regions without apparent lesions on CT, the SUV_mean_ gradually increased from day 7 to day 21, reflecting ongoing fibrotic activity. Moreover, HE staining and Masson’s trichrome staining did not reveal pulmonary fibrosis, while PET imaging was able to detect it, serving the purpose of early diagnosis. At 30 min and 60 min, the target-to-background ratio (TBR) of the PQ groups on day 7, 14, and 21 was significantly higher than the control group, suggesting a high specificity of [^18^F]F-FAPI-42 binding to activated fibroblasts.

**Conclusion:**

[^18^F]F-FAPI-42 PET/CT imaging enables early diagnosis and staging of PQ-induced pulmonary fibrosis, demonstrating its feasibility and potential for characterizing early disease stages.

**Supplementary Information:**

The online version contains supplementary material available at 10.1186/s13550-024-01118-1.

## Introduction

Pulmonary fibrosis is a chronic lung disease with over 200 identified risk factors, encompassing genetic variations, autoimmune diseases, and environmental exposures [1]. Long-term exposure to noxious substances like tobacco smoke or other pollutants (such as Paraquat, PQ) is a classic factor contributing to lung failure [2]. PQ is a highly efficient, cost-effective, and environmentally protective bipyridine quaternary amine herbicide [3]. Its extensive use in China and other Asian countries over recent decades has resulted in an increase in accidental or intentional PQ poisoning cases. PQ poisoning often induces multi-organ failure, primarily affecting the lungs, leading to interstitial inflammation, edema, epithelial-mesenchymal transition, enhanced fibroblast proliferation, and extracellular matrix deposition, culminating in pulmonary fibrosis within days to weeks post-exposure [2, 4]. Clinical presentation of acute PQ poisoning typically involves acute respiratory distress syndrome (ARDS) followed by irreversible pulmonary fibrosis [5]. PQ poisoning exhibits high mortality rates, predominantly affecting males over the age of 50, with a median survival time of 2–4 years post-diagnosis [6], and has resulted in over 1000 reported deaths [7]. Hence, early diagnosis of PQ-induced pulmonary fibrosis is crucial for timely intervention and appropriate treatment.

Current clinical diagnosis of pulmonary fibrosis is mainly based on clinical features, computed tomography (CT) and histology/biopsy. While CT is capable to display morphologic changes of the diseased lung, it occurs relatively late during pulmonary fibrosis only when typical scarring lung tissues are formed and cannot provide disease activity information in pulmonary fibrosis [8, 9]. Histology/biopsy, although capable of demonstrating disease activity, is not an ideal choice due to its limited capacity to obtain only a small amount of lung tissue and its invasive nature, which results in high sampling variability and poses risks to patients [10]. Therefore, there is an urgent need to develop a non-invasive method for diagnosing disease activity in pulmonary fibrosis to halt the continuous progression of the early phase of pulmonary fibrosis.

Positron Emission Tomography (PET) constitutes a sensitive molecular imaging technique that allows for real-time quantification of molecular processes in vivo, without the need for an invasive procedure. This valuable tool in clinical settings offers detailed functional tissue imaging and enables accurate disease characterization, facilitating personalized treatment strategies and enhancing patient management and monitoring [11–13]. Therefore, the combined application of PET (providing functional metabolic information) and CT (providing anatomical structural information) holds promise as an effective tool for early diagnosis of pulmonary fibrosis.

Fibroblast activation protein (FAP), a type II transmembrane serine protease in the dipeptidyl peptidase 4 (DPP4) family, exhibits high expression during tissue remodeling but is absent in normal fibroblasts [14], rendering it an optimal target for small molecule inhibitors in diagnostic and therapeutic development [15]. Radiolabeled quinoline-based fibroblast activation protein inhibitors (FAPIs) demonstrate high binding specificity to FAP and serve as PET radiotracers for non-invasive diagnosis and quantitative evaluation of FAP expression in activated fibroblasts [16, 17]. A previous study has suggested that bleomycin-induced pulmonary fibrosis can be early diagnosed using [^68^Ga]Ga-FAPI-46 [8]. However, the pulmonary fibrosis induced by PQ differs from the traditional bleomycin-induced fibrosis. Bleomycin primarily activates extracellular matrix remodeling and collagen degradation pathways, leading to rapid and often self-limiting fibrotic progression [18–20]. In contrast, PQ initially triggers cell cycle regulation processes upon lung entry, progressing to fatal delayed progressive pulmonary fibrosis [21]. Further research is therefore warranted to investigate the potential of FAPI PET imaging for early detection of PQ-induced pulmonary fibrosis. Furthermore, the lower positron yield (^68^Ga, 89.14% vs. ^18^F, 96.86%) and higher positron energy (^68^Ga, 1,899 keV vs. ^18^F, 633 keV) of ^68^Ga as well as the inferior image spatial resolution of ^68^Ga compared to ^18^F (2.4 mm vs. 1.4 mm in all directions) may impact image quality and diminish diagnostic accuracy, particularly for pulmonary fibrosis, which shows diffuse symptoms in lung tissue, distinguishing it from concentrated tumors [22]. Furthermore, ^18^F, with its routine cyclotron production and biologically suitable half-life of 110 min, stands out as a preferred radiotracer for PET imaging studies [23].

In this study, radiolabeled [^18^F]fluoride FAPI ([^18^F]F-FAPI-42), which has been reported for cancer associated fibroblasts (CAFs) tumor imaging with ongoing clinical trials, was selected to evaluate its potential for non-invasive quantitative assessment and early diagnosis of PQ-induced lung fibrosis.

## Materials and methods

### Radioactive synthesis of [^18^F]F-FAPI-42

Utilizing a biochemical cyclotron accelerator liquid target (Cyclone 18/9, IBA), high-pressure [^18^O]H_2_O (purity exceeding 99.7%) was irradiated with 18 MeV protons to generate carrier-free [^18^F]fluoride. The resulting ^18^F crude solution was passed through an activated QMA column, rinsed with 10 mL of sterile water for injection, dried with nitrogen (N_2_), and subsequently eluted with 0.35 mL of 0.5 mol/L sodium acetate solution. 0.2 mg of FAPI-42 was dissolved in 300 µL of dimethyl sulfoxide (DMSO), followed by the addition of 60 µL of aluminum chloride (AlCl_3_, 2 mmol/L). This mixture was then combined with the eluate containing ^18^F and allowed to react at 105 °C for 15 min. Following this, 3.5 mL of sterile water for injection was introduced into the reaction vial, mixed with N_2_, and the resulting product was passed through an activated HLB column, followed by a wash with 30 mL of sterile water for injection. Subsequently, the product was eluted with 1.5 mL of 50% anhydrous ethanol (EtOH) and diluted with 10 mL of sterile saline. The eluate was filtered and sterilized through a sterile filter (Cathivex ® -GV, 0.22 μm. Merck Millipore Ltd. Tullagreen, Carrigtwohill, Co. Cork, IRELAND), and the final product was stored in a lead container. The radiochemical purity was determined by radio-high performance liquid chromatography (LC-20AT, Shimadzu Corporation, Japan). 0.1% Trifluoroacetic acid (TFA) was used as mobile phase A and acetonitrile as mobile phase B, with a retention time of 7 min. The purity was measured to be ≥ 95%.

### Animal experiments

**Fig. 1:**
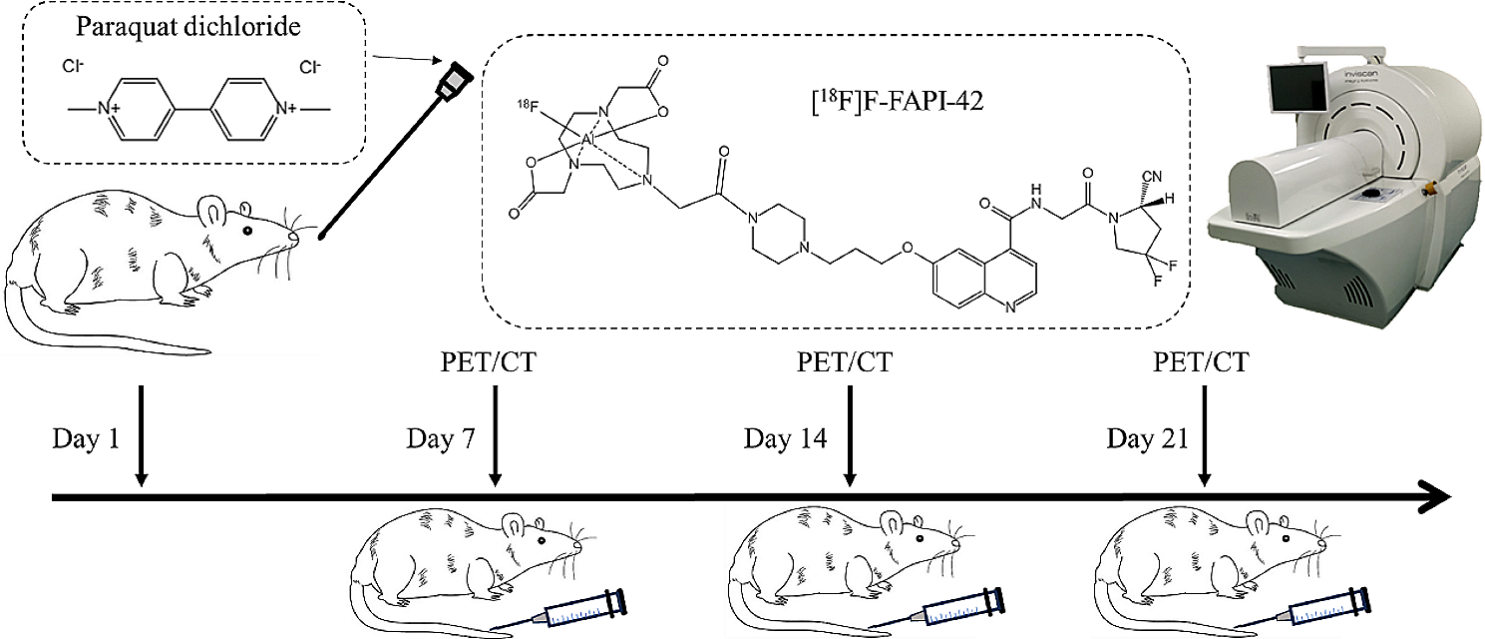
Experimental design of PQ administration and PET/CT imaging. On day 1, paraquat was administered via gavage at a dose of 80 mg/kg. On day 7, 14, and 21, [^18^F]F-FAPI-42 was injected into the tail vein, followed by PET/CT imaging. Rats that were not imaged at each time point were sacrificed

Male 8-week-old SD rats, purchased from the Experimental Animal Center of Southern Medical University, were adaptively fed for 1 week at a temperature of 24 ± 2 °C, with a humidity of 60%±5% and a 12-hour rotation of light and dark. During this period, the rats had free access to food and water, and their feces were promptly removed while observing for any adverse reactions. The PQ powder (with a purity of 85.2%, from Shanghai Ampu Shine Standard Technical Services Co., Ltd.) was diluted in sterile water to a concentration of 10 mg/mL and administered by gavage at a dose of 80 mg/kg body weight. The control group received an equivalent volume of 0.9% saline solution. The general conditions of the rats, including their mental status, diet, and weight changes, were closely monitored. The rats were used for PET/CT imaging studies on day 7, 14, and 21 after intraperitoneal injection. The experimental design is depicted in Fig. 1. All protocols were approved by the Ethics Review Committee of Southern Medical University (L2018198).

### PET/CT imaging

PET/CT scans were acquired using a small animal PET/CT imaging scanner (IRIS PET/CT, Inviscan SAS, Strasbourg, France). The intrinsic axial resolution of PET scanning is 1 mm. Immediate dynamic PET/CT scans were performed for one hour immediately after the injection of [^18^F]F-FAPI-42. PET imaging scans were conducted using three-dimensional ordered-subset expectation maximum-monte carlo (3D-OSEM-MC) algorism with 8 subsets and 8 iterations. The PET imaging frames protocol consisted of 38 frames with the following durations: 4 × 5 s, 6 × 10 s, 5 × 20 s, 4 × 30 s, 5 × 60 s, 5 × 120 s, 5 × 240 s, 4 × 300 s. CT imaging utilized the following parameters: 80 kVp, 1 mA, and a nominal axial resolution of 60 μm. Prior to scanning, the rats received 3-4% isoflurane inhalation anesthesia, and during the dynamic 1-hour PET scan, the anesthesia level was maintained at 1.5-2.5%. After securing the animals on the scanner bed, an intravenous indentation needle was inserted into the tail vein, and 3-3.5 MBq (100–250 µL) [^18^F]F-FAPI-42 were administered slowly. Both experimental and control groups underwent dynamic PET/CT scans throughout the first hour after injecting [^18^F]F-FAPI-42 on day 7, 14, and 21 after PQ administration. After injecting of [^18^F]F-FAPI-42 into the rats, the tracer circulated in the blood and was subsequently absorbed by various tissues and organs. PET/CT images were processed using Imalytics Preclinical software, and the quantitative measurement of the uptake of [^18^F]F-FAPI-42 in the lungs was determined using the mean standardized uptake value (SUV_mean_).

### Elimination of confounding factor

Biological factors, such as blood flow, inflammation, and edema, may contribute to the non-specific uptake of [^18^F]F-FAPI-42. To assess its impact on SUV_mean_, we established a pulmonary fibrosis model in C57BL/6J mice by intraperitoneally injecting 40 mg/kg PQ. The day before PET/CT imaging, we induced sterile inflammation model in the right hind limb gastrocnemius muscle of the mice by injecting 0.1 mL turpentine oil. On the imaging day, [^68^Ga]Ga-FAPI-42 was administered via the tail vein. Calculate if there is a difference in SUV_mean_ between the right and left legs of the mice.

### Histological analysis

Following PET/CT imaging, the rats were euthanized. Their lungs were then fixed in 4% paraformaldehyde overnight. Subsequently, the tissues underwent dehydration using a series of alcohol gradients and were cleared in xylene. Finally, the samples were embedded in paraffin and sectioned at 3 micrometers for hematoxylin-eosin (HE) staining, Masson-trichrome (Masson) staining and NOTA-FAPI-04-MB fluorescent probe staining. We utilized NOTA-FAPI-04-MB for fluorescence staining of paraffin-embedded tissue slices. The 3 μm thick paraffin sections were deparaffinized and rehydrated, blocked with 1% BSA, and then stained with 10 µM NOTA-FAPI-04-MB fluorescence probe for FAP, with DAPI used for nuclear staining. Observation was conducted under a fluorescence microscope (THUNDER Imager 3D, Leica) at 635/700 nm (Excitation/Emission).

### Statistical analysis

All statistical analysis and graphs were performed using Graph Pad Prism 9.0. Data were expressed as mean ± standard deviation (SD). Student’s t-test and one-way analysis of variance (One-way ANOVA) were used for data analysis. A p-value (two-tailed) less than 0.05 was considered statistically significant.

## Results

### General situation of rats

Upon observation, the control group rats displayed lustrous and sleek fur, exhibited active and nimble movements, maintained steady respiration, showed normal feeding behavior, retained stable body weight, and survived until the PET/CT imaging session. In contrast, the experimental group rats manifested signs of toxicity, including dull fur, reduced activity levels, significantly diminished appetite, noticeable weight loss, delayed responses, shortness of breath, and occasional coughing within the initial day post-PQ administration. Nevertheless, by the fourth day, the condition of the experimental group rats gradually ameliorated, with their weight incrementally reaching parity with that of the control group rats.

### Elimination of confounding factors

The SUV_mean_ demonstrated that there was no significant disparity in [^68^Ga]Ga-FAPI-42 uptake between the mice’s left and right legs (1.10 ± 0.61 in the left leg vs. 0.94 ± 0.19 in the right leg at 30 min, p-value = 0.5544; 0.93 ± 0.51 in the left leg vs. 0.82 ± 0.17 in the right leg at 60 min, p-value = 0.6062). Thus, the potential impact of inflammation on [^18^F]F-FAPI-42 binding to fibroblast activation protein was deemed negligible. (See Supplementary Material, Fig. **S1****)**

### PET/CT with [^18^F]F-FAPI-42 uptake

**Fig. 2:**
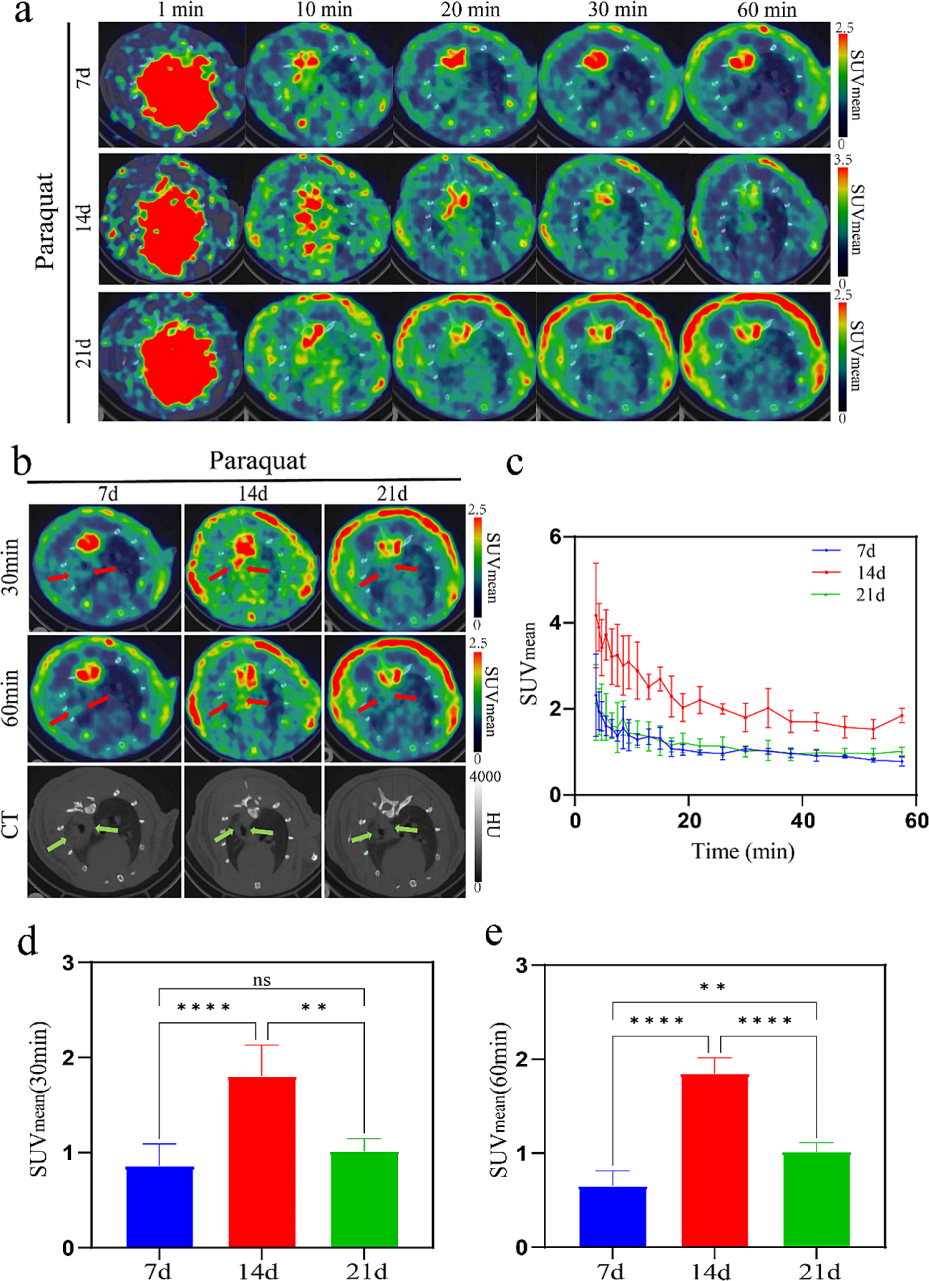
[^18^F]F-FAPI-42 was utilized for dynamic one-hour PET/CT imaging of pulmonary fibrosis induced by PQ in rats, highlighting severe lesion areas on CT scans. (**a**) In the PQ group of rats, the uptake and clearance of [^18^F]F-FAPI-42 during PET/CT imaging on day 7, 14, and 21 were assessed. (**b**) PET/CT fusion images and CT images at 30 min and 60 min, with green arrows indicating severe lesion sites on CT, and red arrows representing PET signals of [^18^F]F-FAPI-42 at severe lesion sites on CT. (**c**) Time-activity curves were generated, with SUV_mean_ values calculated at (**d**) 30 min and (**e**) 60 min. (ns: *P* > 0.05, **: *P* < 0.01, ****: *P* < 0.0001)

The PET/CT images and time-activity curves **(**Fig. 2a, c**)** revealed rapid uptake and slow clearance of the tracer in the lungs across all three imaging dates. In the PQ group, pathological areas were evident in the lung CT scans, with PET signals exhibiting greater concentration on day 7. Subsequent PET/CT scans on day 14 and day 21 revealed a significant increase in PET signal at the site of abnormal lesions on day 14 compared to day 7, followed by a gradual decline on day 21. Time-activity curves **(**Fig. 2c**)** indicated that the tracer uptake in the lungs stabilized around 20 min. Therefore, we opted to compare tracer uptake in the lungs at 30 min and 60 min post-injection to evaluate the intensity of fibrotic activity on day 7, 14, and 21 **(**Fig. 2b, d, e**)**. In regions displaying severe lesions on CT, fibrosis progressed rapidly from day 7 to day 14, with the SUV_mean_ at 30 min being 2.10 times higher on day 14 compared to day 7 (1.80 ± 0.33 vs. 0.86 ± 0.23, p-value < 0.0001). However, from day 14 to day 21, fibrosis progression slowed down, with the SUV_mean_ on day 14 significantly higher than day 21 at 30 min (1.80 ± 0.33 vs. 1.02 ± 0.13, p-value = 0.0013), being 1.77 times higher on day 21. At 60 min, the SUV_mean_ on day 14 was 2.92 times higher than on day 7 (1.85 ± 0.17 vs. 0.63 ± 0.16, p-value < 0.0001), and 1.82 times higher than on day 21 (1.85 ± 0.17 vs. 1.02 ± 0.10, p-value < 0.0001). Furthermore, the SUV_mean_ on day 21 was 1.60 times higher than on day 7 (1.02 ± 0.10 vs. 0.63 ± 0.16, p-value = 0.0044), showing significant statistical differences. This indicates that within one hour after the injection of [^18^F]F-FAPI-42, with increasing imaging time, more tracer bond to fibroblast activation protein in the lungs, while unbound tracer was eliminated.

**Fig. 3:**
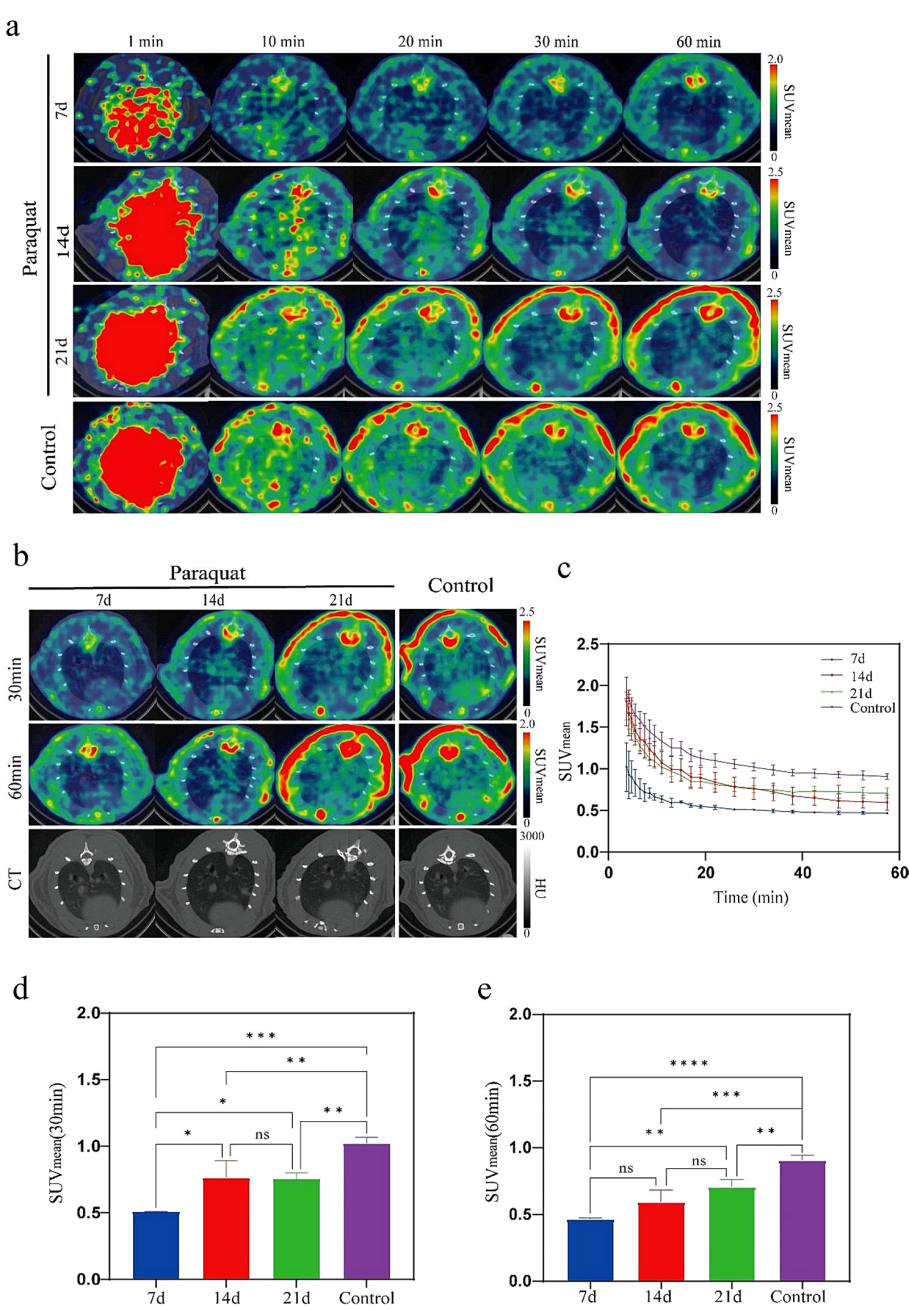
Areas without visible lesions on CT scans. (**a**) The uptake and clearance of [^18^F]F-FAPI-42 during PET/CT imaging within one hour of PQ and control groups. (**b**) PET/CT fusion images and CT images at 30 min and 60 min.(**c**) Time-activity curves were generated, and SUV_mean_ values were calculated at (**d**) 30 min and (**e**) 60 min. (ns: *P* > 0.05, *: *P* < 0.05, **: *P* < 0.01, ***: *P* < 0.001, ****: *P* < 0.0001)

In the PQ group, the normal areas in the lung CT scans exhibited rapid tracer uptake and slow tracer elimination **(**Fig. 3a, c**)**. The uptake of [^18^F]F-FAPI-42 in the lungs gradually increased from day 7 to day 14, and further to day 21. Interestingly, the control group showed higher uptake than the PQ group’s CT non-lesion area. Time-activity curves **(**Fig. 3c**)** revealed a progressive increase in SUV_mean_ from day 7 to day 14, and then to day 21, with the control group’s SUV_mean_ significantly surpassing that of the PQ group. Similarly, we assessed the status of lung fibrotic activity at 30 min and 60 min **(**Fig. 3b, d, e**)**. At 30 min, the tracer uptake on day 14 was significantly higher than on day 7 (0.76 ± 0.13 vs. 0.51 ± 0.01, p-value = 0.0147), while there was no significant difference between day 21 and day 14, but day 21 remained significantly higher than day 7 (0.76 ± 0.04 vs. 0.51 ± 0.01, p-value = 0.0184). At 60 min, there was no significant difference in SUV_mean_ between day 7 and day 14, and between day 14 and day 21, but the SUV_mean_ on day 21 was significantly higher than on day 7 (0.71 ± 0.06 vs. 0.47 ± 0.01, p-value = 0.004), indicating continuous progression of fibrosis from day 7 to day 21. This finding highlights the capability of PET imaging using [^18^F]F-FAPI-42 to detect early fibrotic lesions that remain undetectable on CT scans. As a result, it enables early diagnosis of lung fibrosis. It is worth noting that at 30 min and 60 min, the control group’s SUV_mean_ (1.02 ± 0.05 at 30 min; 0.91 ± 0.04 at 60 min) was significantly higher than that of the PQ group on day 7 (p-value < 0.0001 at 30 min; p-value < 0.0001 at 60 min), day 14 (p-value = 0.0053 at 30 min; p-value = 0.0001 at 60 min), and day 21 (p-value = 0.0041 at 30 min; p-value = 0.0056 at 60 min). We also quantified the SUV_mean_ of [^18^F]F-FAPI-42 uptake in other organs and tissues, including the heart, liver, and kidneys. Time-activity curves revealed that the kidneys exhibited a faster uptake and clearance rate compared to other organs, suggesting that the kidneys is the primary organ for the metabolism of [^18^F]F-FAPI-42. (See Supplementary Material, Fig. **S2)**

**Fig. 4:**
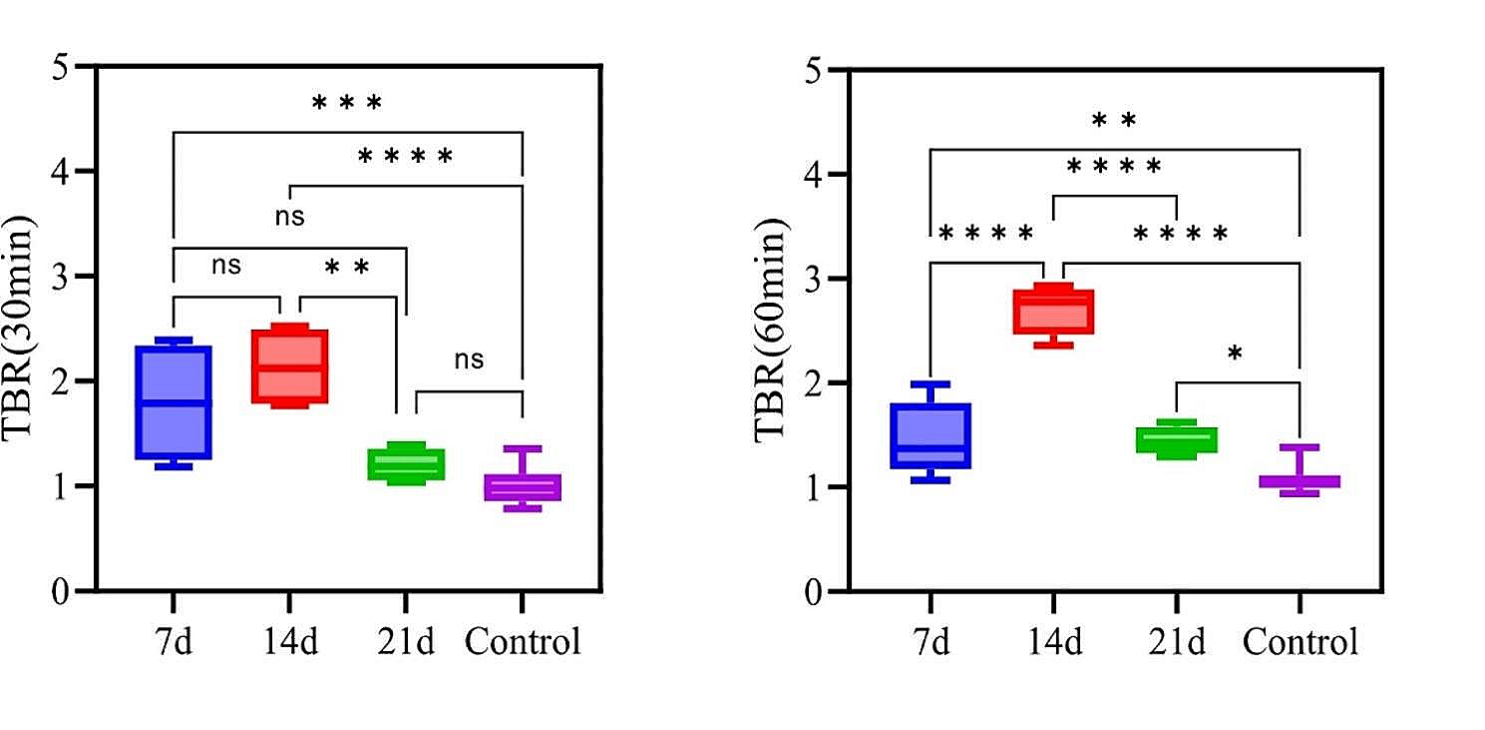
The target-to-background ratio (TBR) values of the lungs of rats in the PQ groups on day 7, 14, and 21, as well as the control group rats, at 30 min and 60 min. (*: *P* < 0.05, **: *P* < 0.01,***: *P* < 0.001, ****: *P* < 0.0001)

We calculated the SUV_mean_ of the PET signal concentration area and the background area for both the experimental and control groups to calculate the target-to-background ratio (TBR). The TBR was used to assess the specific binding of [^18^F]F-FAPI-42 to FAP. The TBR calculation for the experimental group involved selecting a high-uptake lung region in the PQ groups as the target and a corresponding low-uptake region in the control group as the non-target, and then calculating the ratio of target to non-target uptake. The TBR calculation for the control group entailed selecting a lung region corresponding to the high-uptake area in PQ groups as the target, and a low-uptake region in the control group as the non-target, and subsequently computing the ratio of target to non-target uptake. To compare the TBR between the experimental and control groups, we evaluated the data at two time points: 30 min and 60 min **(**Fig. 4**)**. At 30 min, the TBR on day 7 was 1.78 times higher in the control group, with a statistically significant difference (1.78 ± 0.55 vs. 1.00 ± 0.16, p-value = 0.0003). The TBR on day 14 was 2.13 times higher in the control group, also significantly higher than in the control group (2.13 ± 0.39 vs. 1.00 ± 0.16, p-value < 0.0001), while the TBR on day 21 (1.20 ± 0.15 vs. 1.00 ± 0.16, p-value = 0.76) showed no statistically significant difference compared to the control group. At 60 min, the TBR on day 14 significantly increased compared to 30 min (2.71 ± 0.24 vs. 2.13 ± 0.40, p-value = 0.0221). The TBR on day 14 was 1.84 times higher than on day 7 (2.71 ± 0.24 vs. 1.47 ± 0.34, p-value < 0.0001), 1.88 times higher than on day 21 (2.71 ± 0.24 vs. 1.45 ± 0.16, p-value < 0.0001), and 2.50 times higher than in the control group (2.71 ± 0.24 vs. 1.08 ± 0.11, p-value < 0.0001). The TBR on day 7, 14, and 21 was significantly higher than the control group (day 7 vs. control, p-value = 0.0040; day 14 vs. control, p-value < 0.0001; day 21 vs. control, p-value = 0.0441).

### Histological analysis

**Fig. 5:**
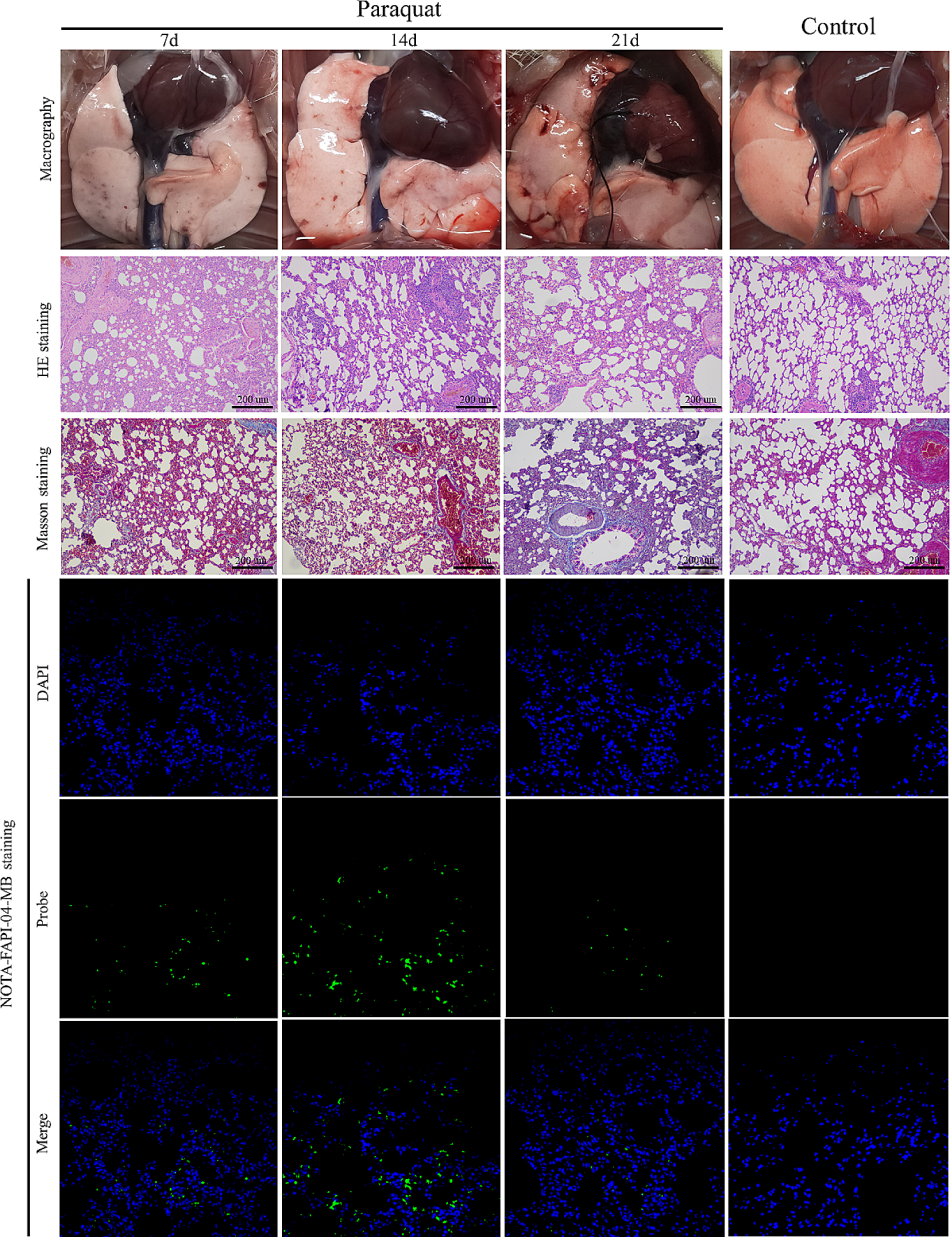
With prolonged PQ poisoning, the ecchymosis and petechiae in the lungs of PQ groups decreased continuously. HE staining indicated a gradual resolution of pulmonary inflammation in PQ groups. Masson’s trichrome staining did not reveal significant fibrotic lesions. The NOTA-FAPI-04-MB fluorescence probe staining showed that FAP expression peaked on day 14

Following completion of the [^18^F]F-FAPI-42 PET/CT scans, all rats were euthanized, and their lungs were harvested for the evaluation of pulmonary fibrosis, including macrography observation and pathological analysis **(**Fig. 5**)**. After gastric administration of PQ, noticeable ecchymosis and petechiae appeared in the lungs of the rats. The number of ecchymosis and petechiae was highest on day 7, decreased on day 14, and further reduced on day 21. In contrast, the lungs of the control group rats appeared redder without ecchymosis or petechiae. HE staining revealed alveolar interval thickening, infiltration of inflammatory cells, and a gradual reduction in lung inflammation over time. Masson’s trichrome staining did not show any significant fibrosis. Notably, NOTA-FAPI-04-MB fluorescence probe staining showed that on day 14, FAP expression peaked, indicating the highest fibrotic activity following paraquat gavage.

## Discussion

Despite the application of various treatment modalities, PQ-induced pulmonary fibrosis is the leading cause of mortality in individuals who have accidentally ingested PQ [24]. Consequently, early diagnosis of fibrosis progression and adjustment of treatment approaches become imperative in ensuring patient recovery. For early diagnosis, CT and histology/biopsy exhibit limitations. In contrast, PET/CT, routinely utilized for clinical diagnoses including cancer, cardiovascular, and neuroimaging, offers significant advantages in early diagnosis due to its molecular-level insights and quantitative capabilities [25]. However, conventional clinical [^18^F]F-FDG PET/CT imaging is limited to detecting inflammation and does not allow for the detection of the dynamic tissue destruction process [26]. Developing targeted probes for fibrosis-related FAP targets presents an intriguing avenue. [^68^Ga]Ga-FAPI-46 has demonstrated efficacy in assessing bleomycin-induced idiopathic pulmonary fibrosis by targeting highly expressed FAP cells, showing promising applications [8]. Bleomycin induces cellular necrosis and apoptosis, inflammation, and fibrosis via DNA strand breakage, free radical generation, oxidation of fatty acids, and destabilization of membranes [18, 27]. Pulmonary fibrosis induced by bleomycin is typically self-limiting, with potential rapid recovery and partial return to normalcy [20]. In contrast, the mechanism underlying pulmonary fibrosis induced by PQ poisoning is primarily associated with the redox cycle after paraquat enters the cells, although it’s not fully understood [28, 29]. Early stages may include excessive reactive oxygen species (ROS) and PQ radicals, damaging alveolar epithelial cells and activating inflammation. Later stages involve fibroblast proliferation, alveolar structure destruction, and progressive fibrosis [21, 27, 30]. The diverse mechanisms underlying pulmonary fibrosis induced by bleomycin and PQ can result in distinct impacts and pathological outcomes. Therefore, further research is needed for early diagnosis of lung fibrosis induced by PQ. With its superior nuclide properties, ^18^F is widely used in clinical settings, rendering [^18^F]F-labeled FAPI a preferred option for pulmonary fibrosis diagnosis. This study evaluates the potential of [^18^F]F-FAPI-42 for another type of pulmonary fibrosis induced by PQ.

Inflammation is one of the key triggering factors for fibrosis, and the inflammatory response marks the initiation stage of the fibrotic process [31]. When lung tissue is damaged or infected, it activates immune cells and prompts the release of inflammatory mediators, which can lead to a robust fibrotic response. Fibroblasts, stimulated by inflammatory mediators, proliferate and synthesize collagen, ultimately leading to the formation of fibrotic lesions [32]. The tracer uptake at the site of inflammation in the mice’s gastrocnemius muscle showed no difference compared to the unaffected leg, ruling out non-specific uptake due to inflammation and edema. This suggests that inflammation and edema didn’t interfere with the binding of [^18^F]F-FAPI-42 to fibroblast activation protein, enhancing the specificity of [^18^F]F-FAPI-42. In areas with evident lesions on CT scans, significant tissue damage was observed, indicating a potentially severe inflammatory response. Due to the high severity of the lesions, the fibrotic process may already be in an active stage. The SUV_mean_ values indicats that the uptake of [^18^F]F-FAPI-42 at 30 min and 60 min on day 14 was significantly higher than on days 7 and 21. This suggests that the fibrotic process gradually intensified by day 7, peaked on day 14, and then decreased by day 21 due to the transition into inactive fibrotic areas. This highlights that PET/CT imaging can assess fibrotic activity status based on the uptake of [^18^F]F-FAPI-42 in lung fibrotic lesion areas visualized on CT scans. It provides quantitative information about metabolic activity in these areas, enabling staging of pulmonary fibrosis disease activity and assisting clinicians in more accurately assessing disease progression and severity. In PET images of areas without evident lesions on CT, the tracer uptake on day 21 was significantly higher than on day 7. Although there was no significant statistical difference in SUV_mean_ between days 14 and 21, a gradual increase in SUV_mean_ at 60 min in areas without evident lesions on CT suggests a trend of increasing fibrotic activity. We can observe that, compared to the areas with evident lesions on CT scans, regions without apparent lesions on CT exhibited lower fibrotic activity and ongoing fibrotic progression. This suggests that areas without visible lesions on CT might contain low-level inflammation and fibrotic activity that have not yet led to significant structural changes, potentially requiring more time to manifest noticeable alterations. In scenarios where early diagnosis is challenging with CT imaging alone, utilizing [^18^F]F-FAPI-42 for PET imaging enables more precise detection of early signs of fibrotic activity, facilitating early diagnosis of pulmonary fibrosis. The varying patterns of fibrotic activity in areas with and without evident lesions on CT at different imaging dates reflected the dynamic balance and regulation between inflammation and fibrotic activity. This approach allowed for staging of pulmonary fibrosis and early diagnosis by capturing the evolving fibrotic activity in distinct regions on CT.

Furthermore, we observed an intriguing phenomenon where tracer uptake in the control group was significantly higher than in the PQ group. We believe that areas with evident lesions on CT scans indicate more severe damage and higher fibrotic activity. When a fixed dose of tracer wass administered, more tracer bond to the areas with evident lesions on CT scans, i.e. where there was a concentrated and visible uptake on the PET image. In regions where CT scans didn’t show evident lesions, tracer distribution was relatively low. In addition, a previous studies have shown that rats poisoned with paraquat exhibit endothelial damage, leading to localized microthrombus formation due to exposed endothelium. This affects pulmonary blood flow and volume, resulting in decreased pulmonary blood flow rate and intravascular blood volume in lung tissues [33]. In comparison to the control group, where the lungs are not affected by inflammation or other pathophysiological processes, blood circulation is smoother, with higher perfusion and blood flow rates. Consequently, healthy lung tissues exhibit higher blood content, leading to increased tracer allocation in these tissues. As a result, there is a more uniform distribution of the tracer in the lungs, potentially enhancing the contribution of blood signals to the overall lung signal [34]. For instance, in the bleomycin-infused rat model of lung fibrosis, the control group exhibits a more uniform distribution of blood flow, resulting in a greater distribution of ^99m^Tc-PulmoBind endothelial tracer in the lungs [35]. In our study, the blood flow rates and volume in the lung blood vessels of the experimental group were affected by paraquat-induced endothelial damage. As a result, less tracer was received compared to the control group, leading to lower tracer uptake in regions without evident lesions on CT scans in the experimental group, which is a reasonable observation. Overall, the dynamic changes in fibrotic activity may vary between regions with evident CT lesions and those without. These variations could be influenced by the degree of inflammation and tissue damage, leading to differing intensities and speeds of the fibrotic process at different time points. In addition, to investigate the specificity of [^18^F]F-FAPI-42 binding to fibroblast activation protein and its performance in early diagnosis of pulmonary fibrosis, we calculated the target-to-background ratio (TBR). The results indicated that at 60 min, the TBR on day 14 was significantly higher than on day 7 and 21, suggesting peak fibrotic activity on day 14, with no significant difference between day 7 and 21, consistent with the PET signal intensity in areas with evident CT lesions. Additionally, in the PQ group, the TBR on day 7, 14, and 21 was significantly higher than in the control group, indicating the specific binding of [^18^F]F-FAPI-42 to fibroblast activation protein during the fibrotic process.

In general, rats exhibited decreased activity and deteriorated mental status within four days after gastric lavage with PQ. However, their condition began to improve after four days. Subsequently, rats in the PQ group showed a gradual increase in body weight and improvement in their condition, approaching that of the control group, mirroring the pulmonary phenotype of the rats. On day 7, distinct ecchymosis and spots appeared on the lung surface induced by PQ, which decreased by day 14 and further reduced by day 21, with the lung color gradually becoming rosier, resembling that of the control group. However, intriguingly, PET images revealed that the peak of the fibrotic process in rats occurred after their condition started to improve, suggesting that the clinical symptoms of the disease may not entirely align with the underlying pathophysiological processes.

The improvement in the rats’ general condition after the fourth day might be attributed to a reduction in systemic inflammatory response or compensatory mechanisms, rather than necessarily indicating a reduction in the severity of pulmonary fibrosis. PET/CT imaging proved sensitive in detecting subtle changes in the fibrotic pathological process, even as the general condition of the rats is improving. This allowed for the observation of a gradual worsening of fibrosis, consistent with the results of HE staining. While inflammation was more severe on day 7, with increased thickening of alveolar septa and infiltration of inflammatory cells, these effects diminished by days 14 and 21. Surprisingly, Masson’s trichrome staining did not reveal significant fibrotic lesions. It’s possible that the sampling location did not display fibrosis visibly at the observed time points, as fibrosis is a progressive process that may not manifested visibly in early or minor stages. Nevertheless, metabolic activity changes were already detectable in PET images. Building upon our previous work demonstrating the excellent binding properties of NOTA-FAPI-04-MB to FAP [36], staining with the NOTA-FAPI-04-MB probe showed FAP expression. From this perspective, the integration of PET with CT offers valuable insights into early disease development, enhancing the accuracy of pulmonary fibrosis diagnosis and treatment. Therefore, this study employing [^18^F]F-FAPI-42 holds significant importance for improving diagnostic precision and treatment effectiveness in PQ-induced pulmonary fibrosis. However, early events may impact the development of subsequent pathological processes, and little is known about how the oxidative signal of PQ interacts with the pathways of pulmonary fibrosis. Further research into the mechanisms by which PQ-induced pulmonary fibrosis can provide a deeper understanding for diagnosis and treatment. Therefore, in further research, we plan to explore the mechanism of PQ-induced pulmonary fibrosis and assess the toxicity of PQ on the pharmacokinetics of radiopharmaceuticals.

## Conclusion

The expression of fibroblast activation protein is strongly correlated with the progression of PQ-induced pulmonary fibrosis and represents a superior target for the early diagnosis and staging of pulmonary fibrosis using [^18^F]F-FAPI-42 PET/CT imaging. These findings emphasize the potential of [^18^F]F-FAPI-42 in enabling early diagnosis and staging of PQ-induced pulmonary fibrosis, thereby assisting in intervention strategies to mitigate its progression.

### Electronic supplementary material

Below is the link to the electronic supplementary material.

Supplementary Material 1

## Data Availability

The datasets used and/or analyzed during the current study are available from the corresponding author on reasonable request.
