# Supplementary material for: Early diagnosis and staging of paraquat-induced pulmonary fibrosis using [18F]F-FAPI-42 PET/CT imaging
Source: EJNMMI Res. 2024 Jun 18;14:57. doi: 10.1186/s13550-024-01118-1 (PMC11189367; doi:10.1186/s13550-024-01118-1)
Supplement: Supplementary file 1 — Supplementary Material 1 [file 13550_2024_1118_MOESM1_ESM.docx]

**Supplementary materials**


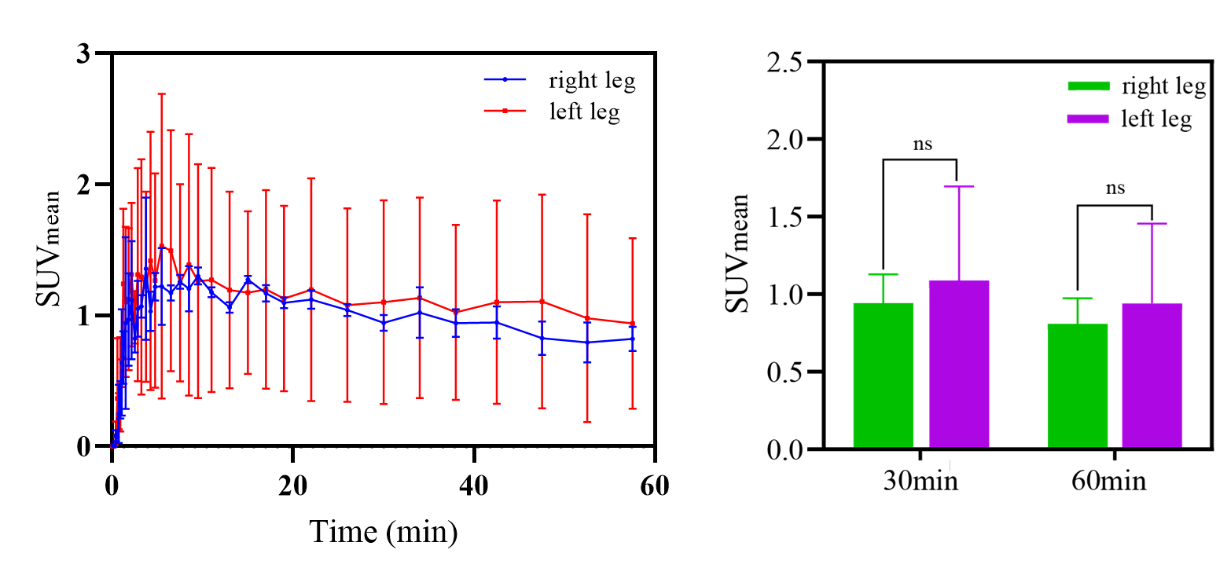


Fig. S1 In the inflammation model of mice, turpentine oil was injected into the gastrocnemius muscle of the right hind leg, and the uptake of [^68^Ga]Ga-FAPI-42 in the left and right legs was assessed.


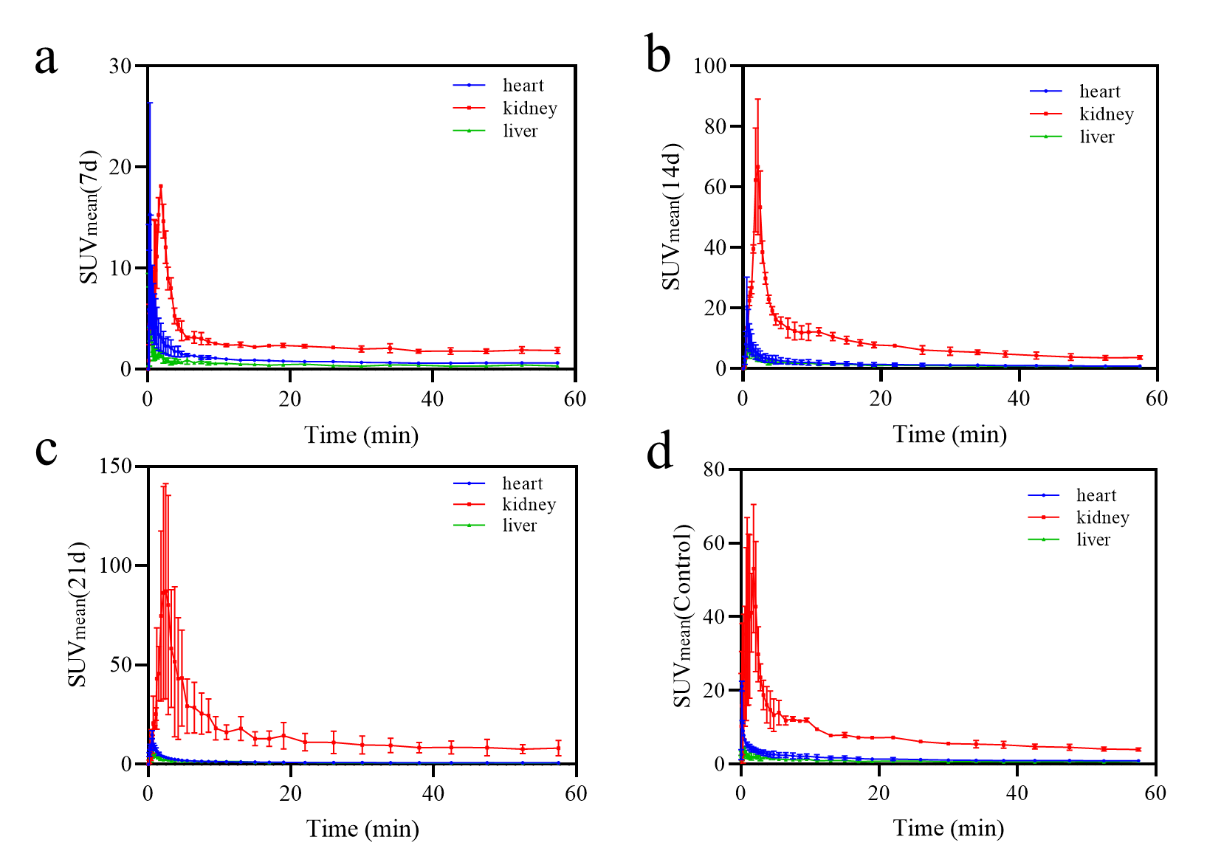


Fig. S2 The uptake of [^18^F]F-FAPI-42 in the heart, liver, and kidneys of rats in the experimental groups on days 7 (a), 14 (b), and 21 (c), as well as in the control group (d).
